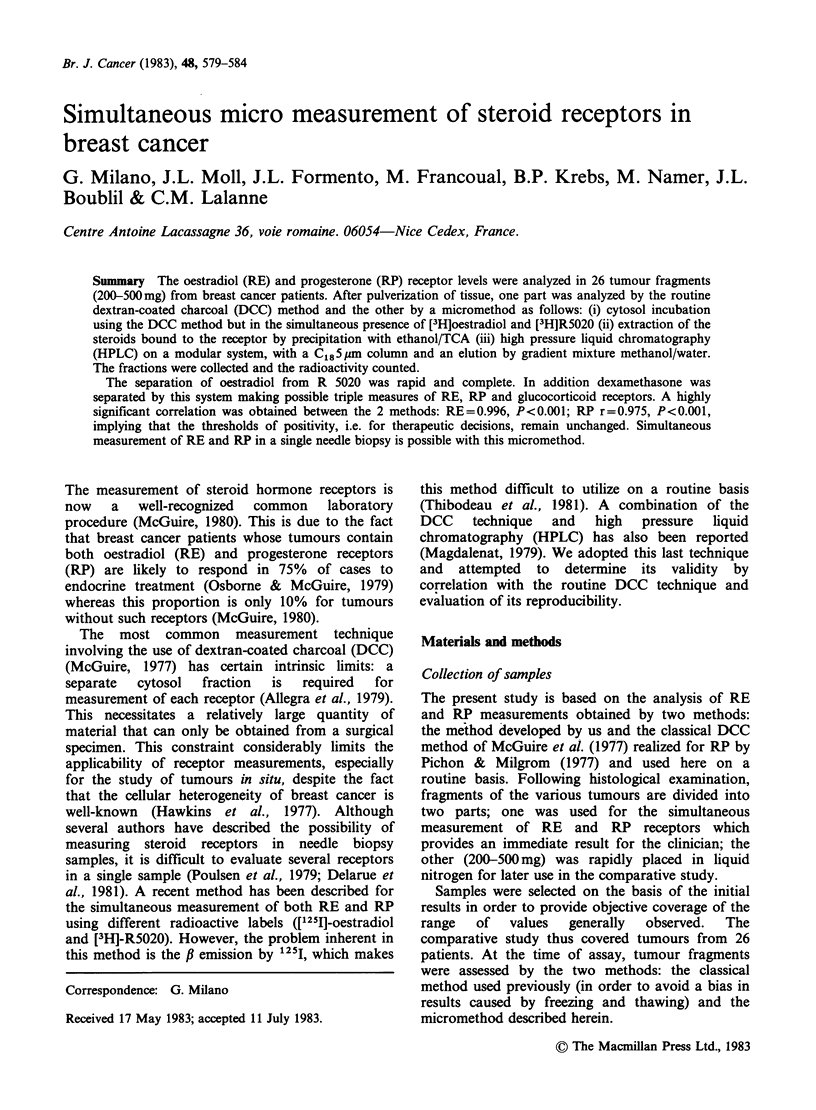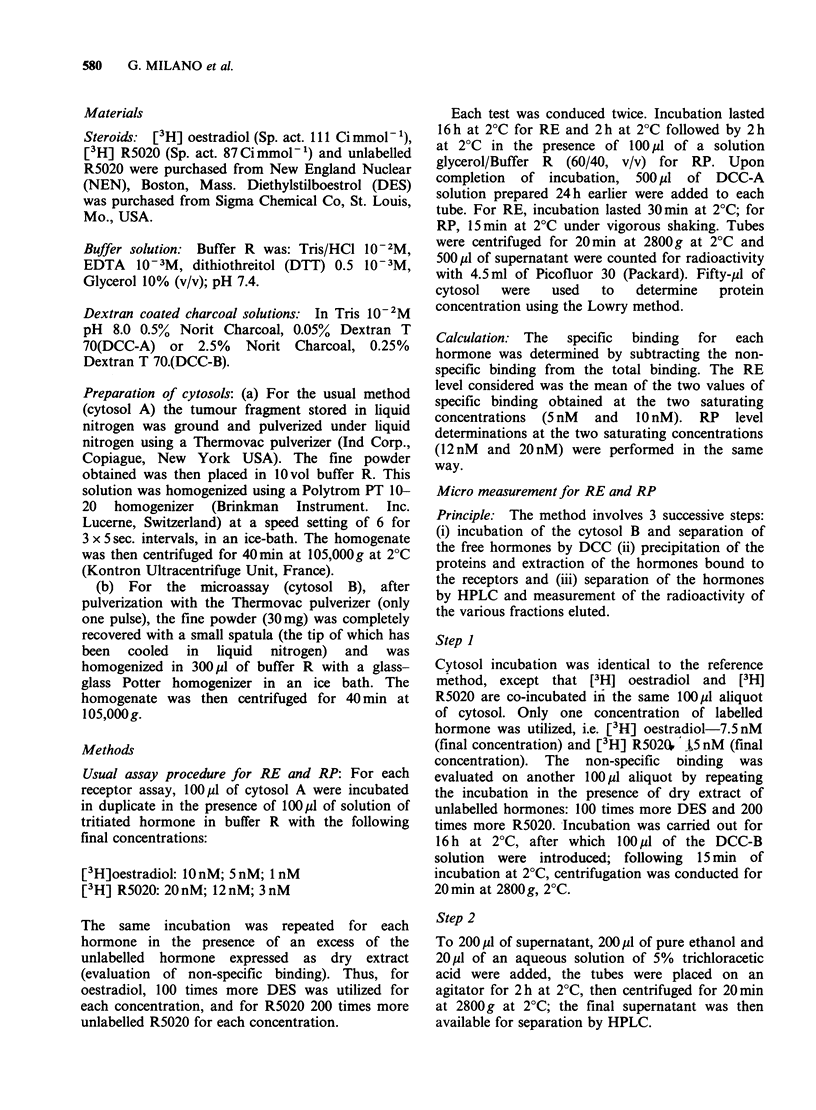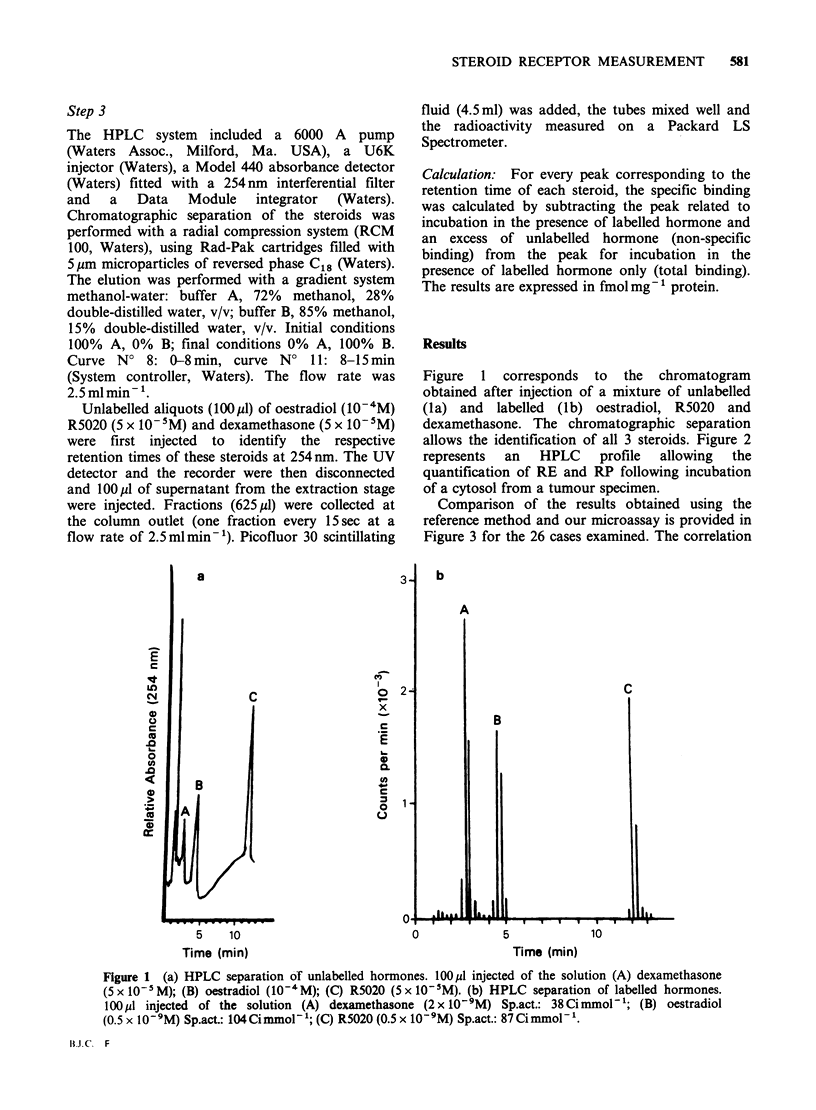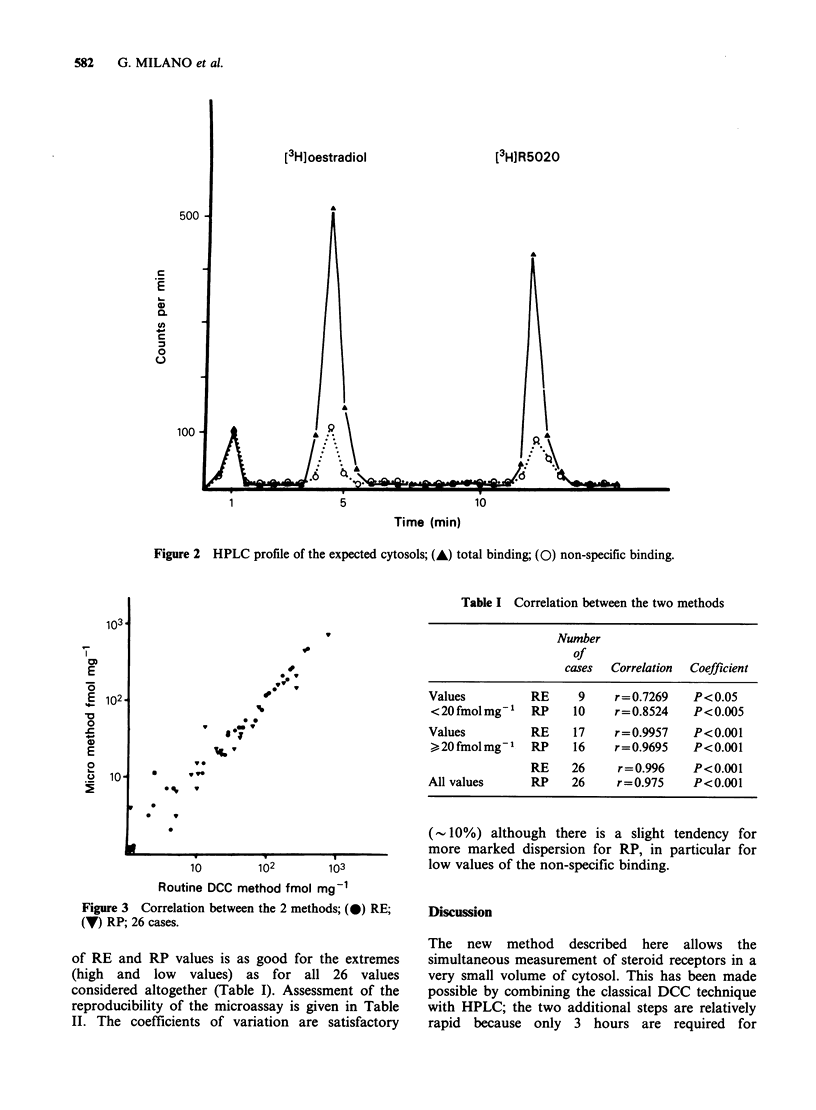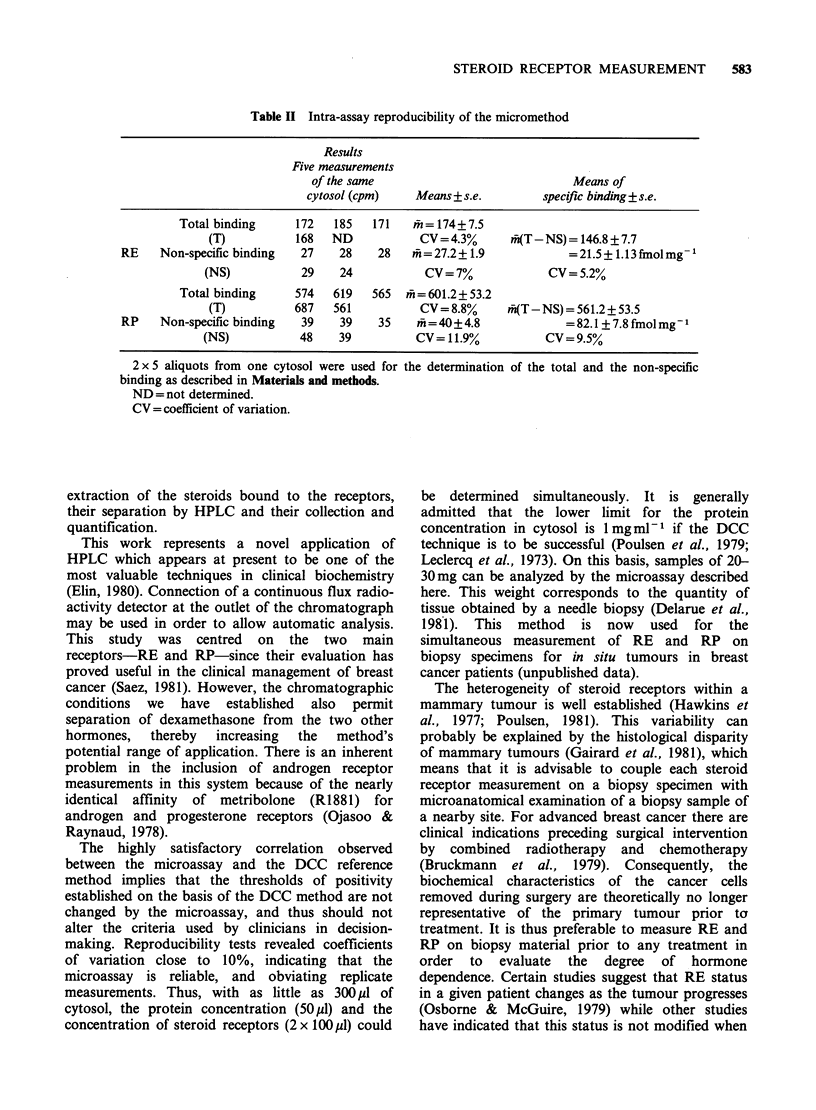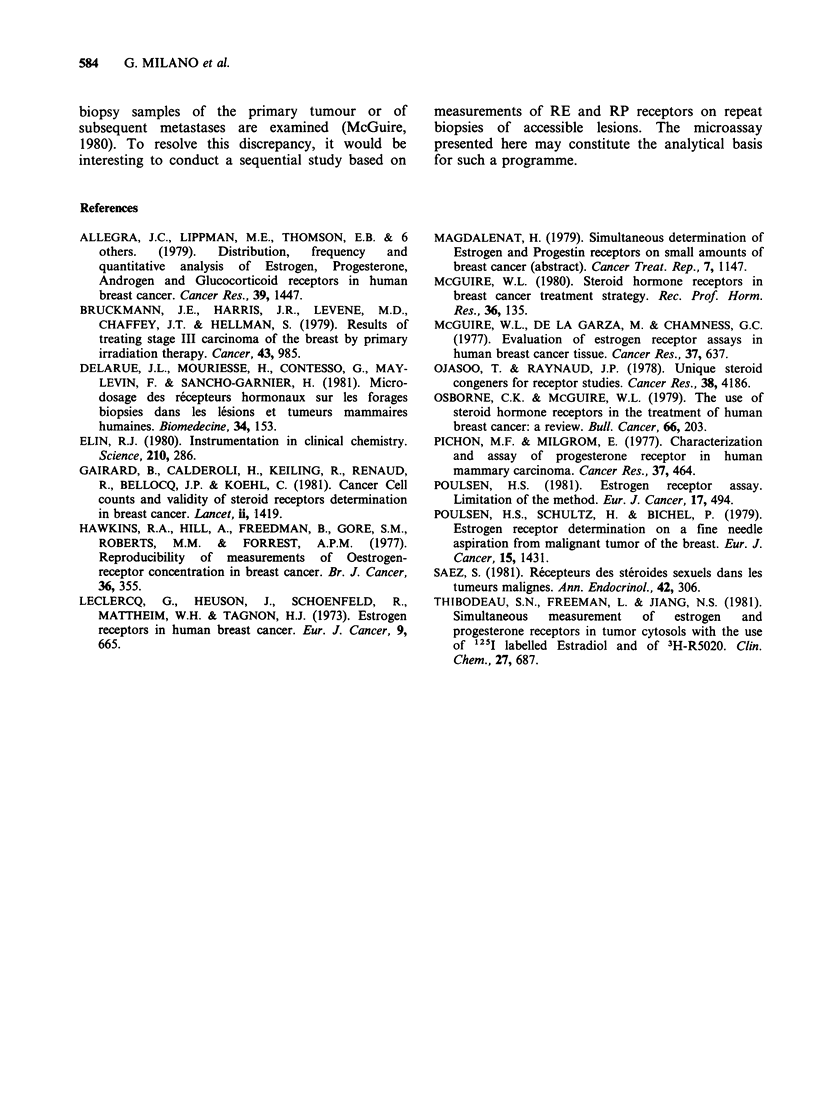# Simultaneous micro measurement of steroid receptors in breast cancer.

**DOI:** 10.1038/bjc.1983.231

**Published:** 1983-10

**Authors:** G. Milano, J. L. Moll, J. L. Formento, M. Francoual, B. P. Krebs, M. Namer, J. L. Boublil, C. M. Lalanne

## Abstract

The oestradiol (RE) and progesterone (RP) receptor levels were analyzed in 26 tumour fragments (200-500 mg) from breast cancer patients. After pulverization of tissue, one part was analyzed by the routine dextran-coated charcoal (DCC) method and the other by a micromethod as follows: (i) cytosol incubation using the DCC method but in the simultaneous presence of [3H]oestradiol and [3H]R5020 (ii) extraction of the steroids bound to the receptor by precipitation with ethanol/TCA (iii) high pressure liquid chromatography (HPLC) on a modular system, with a C185 microns column and an elution by gradient mixture methanol/water. The fractions were collected and the radioactivity counted. The separation of oestradiol from R 5020 was rapid and complete. In addition dexamethasone was separated by this system making possible triple measures of RE, RP and glucocorticoid receptors. A highly significant correlation was obtained between the 2 methods: RE = 0.996, P less than 0.001; RP r = 0.975, P less than 0.001, implying that the thresholds of positivity, i.e. for therapeutic decisions, remain unchanged. Simultaneous measurement of RE and RP in a single needle biopsy is possible with this micromethod.


*Br. J. Cancer* (1983), **48**, 579–584

### Simultaneous micro measurement of steroid receptors in breast cancer

G. Milano, J.L. Moll, J.L. Formento, M. Francoual, B.P. Krebs, M. Namer, J.L. Boublil & C.M. Lalanne

*Centre Antoine Lacassagne 36, voie romaine. 06054—Nice Cedex, France.*

**Summary** The oestradiol (RE) and progesterone (RP) receptor levels were analyzed in 26 tumour fragments (200–500 mg) from breast cancer patients. After pulverization of tissue, one part was analyzed by the routine dextran-coated charcoal (DCC) method and the other by a micromethod as follows: (i) cytosol incubation using the DCC method but in the simultaneous presence of [$^3$H]oestradiol and [$^3$H]R5020 (ii) extraction of the steroids bound to the receptor by precipitation with ethanol/TCA (iii) high pressure liquid chromatography (HPLC) on a modular system, with a $C_{18}$ 5 $\mu$m column and an elution by gradient mixture methanol/water. The fractions were collected and the radioactivity counted.

The separation of oestradiol from R 5020 was rapid and complete. In addition dexamethasone was separated by this system making possible triple measures of RE, RP and glucocorticoid receptors. A highly significant correlation was obtained between the 2 methods: RE=0.996, $P<0.001$; RP $r=0.975$, $P<0.001$, implying that the thresholds of positivity, i.e. for therapeutic decisions, remain unchanged. Simultaneous measurement of RE and RP in a single needle biopsy is possible with this micromethod.

The measurement of steroid hormone receptors is now a well-recognized common laboratory procedure (McGuire, 1980). This is due to the fact that breast cancer patients whose tumours contain both oestradiol (RE) and progesterone receptors (RP) are likely to respond in 75% of cases to endocrine treatment (Osborne & McGuire, 1979) whereas this proportion is only 10% for tumours without such receptors (McGuire, 1980).

The most common measurement technique involving the use of dextran-coated charcoal (DCC) (McGuire, 1977) has certain intrinsic limits: a separate cytosol fraction is required for measurement of each receptor (Allegra *et al.*, 1979). This necessitates a relatively large quantity of material that can only be obtained from a surgical specimen. This constraint considerably limits the applicability of receptor measurements, especially for the study of tumours *in situ,* despite the fact that the cellular heterogeneity of breast cancer is well-known (Hawkins *et al.*, 1977). Although several authors have described the possibility of measuring steroid receptors in needle biopsy samples, it is difficult to evaluate several receptors in a single sample (Poulsen *et al.*, 1979; Delarue *et al.*, 1981). A recent method has been described for the simultaneous measurement of both RE and RP using different radioactive labels ([$^{125}$I]-oestradiol and [$^3$H]-R5020). However, the problem inherent in this method is the $\beta$ emission by $^{125}$I, which makes this method difficult to utilize on a routine basis (Thibodeau *et al.*, 1981). A combination of the DCC technique and high pressure liquid chromatography (HPLC) has also been reported (Magdalenat, 1979). We adopted this last technique and attempted to determine its validity by correlation with the routine DCC technique and evaluation of its reproducibility.

Correspondence: G. Milano

Received 17 May 1983; accepted 11 July 1983.

### Materials and methods

### *Collection of samples*

The present study is based on the analysis of RE and RP measurements obtained by two methods: the method developed by us and the classical DCC method of McGuire *et al.* (1977) realized for RP by Pichon & Milgrom (1977) and used here on a routine basis. Following histological examination, fragments of the various tumours are divided into two parts; one was used for the simultaneous measurement of RE and RP receptors which provides an immediate result for the clinician; the other (200–500 mg) was rapidly placed in liquid nitrogen for later use in the comparative study.

Samples were selected on the basis of the initial results in order to provide objective coverage of the range of values generally observed. The comparative study thus covered tumours from 26 patients. At the time of assay, tumour fragments were assessed by the two methods: the classical method used previously (in order to avoid a bias in results caused by freezing and thawing) and the micromethod described herein.

© The Macmillan Press Ltd., 1983

*Materials*

*Steroids:* [$^3$H] oestradiol (Sp. act. 111 Ci mmol$^{-1}$), [$^3$H] R5020 (Sp. act. 87 Ci mmol$^{-1}$) and unlabelled R5020 were purchased from New England Nuclear (NEN), Boston, Mass. Diethylstilboestrol (DES) was purchased from Sigma Chemical Co, St. Louis, Mo., USA.

*Buffer solution:* Buffer R was: Tris/HCl $10^{-2}$M, EDTA $10^{-3}$M, dithiothreitol (DTT) 0.5 $10^{-3}$M, Glycerol 10% (v/v); pH 7.4.

*Dextran coated charcoal solutions:* In Tris $10^{-2}$M pH 8.0 0.5% Norit Charcoal, 0.05% Dextran T 70(DCC-A) or 2.5% Norit Charcoal, 0.25% Dextran T 70.(DCC-B).

*Preparation of cytosols*: (a) For the usual method (cytosol A) the tumour fragment stored in liquid nitrogen was ground and pulverized under liquid nitrogen using a Thermovac pulverizer (Ind Corp., Copiague, New York USA). The fine powder obtained was then placed in 10 vol buffer R. This solution was homogenized using a Polytrom PT 10–20 homogenizer (Brinkman Instrument. Inc. Lucerne, Switzerland) at a speed setting of 6 for 3 x 5 sec. intervals, in an ice-bath. The homogenate was then centrifuged for 40 min at 105,000 *g* at 2°C (Kontron Ultracentrifuge Unit, France).

(b) For the microassay (cytosol B), after pulverization with the Thermovac pulverizer (only one pulse), the fine powder (30 mg) was completely recovered with a small spatula (the tip of which has been cooled in liquid nitrogen) and was homogenized in 300 $\mu$l of buffer R with a glass–glass Potter homogenizer in an ice bath. The homogenate was then centrifuged for 40 min at 105,000 *g*.

*Methods*

*Usual assay procedure for RE and RP*: For each receptor assay, 100 $\mu$l of cytosol A were incubated in duplicate in the presence of 100 $\mu$l of solution of tritiated hormone in buffer R with the following final concentrations:

[$^3$H]oestradiol: 10 nM; 5 nM; 1 nM
[$^3$H] R5020: 20 nM; 12 nM; 3 nM

The same incubation was repeated for each hormone in the presence of an excess of the unlabelled hormone expressed as dry extract (evaluation of non-specific binding). Thus, for oestradiol, 100 times more DES was utilized for each concentration, and for R5020 200 times more unlabelled R5020 for each concentration.

Each test was conduced twice. Incubation lasted 16 h at 2°C for RE and 2 h at 2°C followed by 2 h at 2°C in the presence of 100 $\mu$l of a solution glycerol/Buffer R (60/40, v/v) for RP. Upon completion of incubation, 500 $\mu$l of DCC-A solution prepared 24 h earlier were added to each tube. For RE, incubation lasted 30 min at 2°C; for RP, 15 min at 2°C under vigorous shaking. Tubes were centrifuged for 20 min at 2800 *g* at 2°C and 500 $\mu$l of supernatant were counted for radioactivity with 4.5 ml of Picofluor 30 (Packard). Fifty-$\mu$l of cytosol were used to determine protein concentration using the Lowry method.

*Calculation:* The specific binding for each hormone was determined by subtracting the non-specific binding from the total binding. The RE level considered was the mean of the two values of specific binding obtained at the two saturating concentrations (5 nM and 10 nM). RP level determinations at the two saturating concentrations (12 nM and 20 nM) were performed in the same way.

*Micro measurement for RE and RP*

*Principle:* The method involves 3 successive steps: (i) incubation of the cytosol B and separation of the free hormones by DCC (ii) precipitation of the proteins and extraction of the hormones bound to the receptors and (iii) separation of the hormones by HPLC and measurement of the radioactivity of the various fractions eluted.

*Step 1*

Cytosol incubation was identical to the reference method, except that [$^3$H] oestradiol and [$^3$H] R5020 are co-incubated in the same 100 $\mu$l aliquot of cytosol. Only one concentration of labelled hormone was utilized, i.e. [$^3$H] oestradiol—7.5 nM (final concentration) and [$^3$H] R5020, 15 nM (final concentration). The non-specific binding was evaluated on another 100 $\mu$l aliquot by repeating the incubation in the presence of dry extract of unlabelled hormones: 100 times more DES and 200 times more R5020. Incubation was carried out for 16 h at 2°C, after which 100 $\mu$l of the DCC-B solution were introduced; following 15 min of incubation at 2°C, centrifugation was conducted for 20 min at 2800 *g*, 2°C.

*Step 2*

To 200 $\mu$l of supernatant, 200 $\mu$l of pure ethanol and 20 $\mu$l of an aqueous solution of 5% trichloracetic acid were added, the tubes were placed on an agitator for 2 h at 2°C, then centrifuged for 20 min at 2800 *g* at 2°C; the final supernatant was then available for separation by HPLC.

*Step 3*

The HPLC system included a 6000 A pump (Waters Assoc., Milford, Ma. USA), a U6K injector (Waters), a Model 440 absorbance detector (Waters) fitted with a 254 nm interferential filter and a Data Module integrator (Waters). Chromatographic separation of the steroids was performed with a radial compression system (RCM 100, Waters), using Rad-Pak cartridges filled with 5 $\mu$m microparticles of reversed phase $C_{18}$ (Waters). The elution was performed with a gradient system methanol-water: buffer A, 72% methanol, 28% double-distilled water, v/v; buffer B, 85% methanol, 15% double-distilled water, v/v. Initial conditions 100% A, 0% B; final conditions 0% A, 100% B. Curve N° 8: 0–8 min, curve N° 11: 8–15 min (System controller, Waters). The flow rate was 2.5 ml $min^{-1}$.

Unlabelled aliquots (100 $\mu$l) of oestradiol ($10^{-4}$M) R5020 ($5 \times 10^{-5}$M) and dexamethasone ($5 \times 10^{-5}$M) were first injected to identify the respective retention times of these steroids at 254 nm. The UV detector and the recorder were then disconnected and 100 $\mu$l of supernatant from the extraction stage were injected. Fractions (625 $\mu$l) were collected at the column outlet (one fraction every 15 sec at a flow rate of 2.5 ml $min^{-1}$). Picofluor 30 scintillating fluid (4.5 ml) was added, the tubes mixed well and the radioactivity measured on a Packard LS Spectrometer.

*Calculation:* For every peak corresponding to the retention time of each steroid, the specific binding was calculated by subtracting the peak related to incubation in the presence of labelled hormone and an excess of unlabelled hormone (non-specific binding) from the peak for incubation in the presence of labelled hormone only (total binding). The results are expressed in fmol $mg^{-1}$ protein.

### Results

Figure 1 corresponds to the chromatogram obtained after injection of a mixture of unlabelled (1a) and labelled (1b) oestradiol, R5020 and dexamethasone. The chromatographic separation allows the identification of all 3 steroids. Figure 2 represents an HPLC profile allowing the quantification of RE and RP following incubation of a cytosol from a tumour specimen.

Comparison of the results obtained using the reference method and our microassay is provided in Figure 3 for the 26 cases examined. The correlation

**Figure 1** (a) HPLC separation of unlabelled hormones. 100 $\mu$l injected of the solution (A) dexamethasone ($5 \times 10^{-5}$ M); (B) oestradiol ($10^{-4}$ M); (C) R5020 ($5 \times 10^{-5}$M). (b) HPLC separation of labelled hormones. 100 $\mu$l injected of the solution (A) dexamethasone ($2 \times 10^{-9}$M) Sp.act.: 38 Ci $mmol^{-1}$; (B) oestradiol ($0.5 \times 10^{-9}$M) Sp.act.: 104 Ci $mmol^{-1}$; (C) R5020 ($0.5 \times 10^{-9}$M) Sp.act.: 87 Ci $mmol^{-1}$.

**Figure 2** HPLC profile of the expected cytosols; (▲) total binding; (○) non-specific binding.

**Figure 3** Correlation between the 2 methods; (●) RE; (▼) RP; 26 cases.

of RE and RP values is as good for the extremes (high and low values) as for all 26 values considered altogether (Table I). Assessment of the reproducibility of the microassay is given in Table II. The coefficients of variation are satisfactory

**Table I** Correlation between the two methods

| | | *Number of cases* | *Correlation* | *Coefficient* |
|---|---|---|---|---|
| Values $<20$ fmol mg$^{-1}$ | RE | 9 | $r=0.7269$ | $P<0.05$ |
| | RP | 10 | $r=0.8524$ | $P<0.005$ |
| Values $\geqslant 20$ fmol mg$^{-1}$ | RE | 17 | $r=0.9957$ | $P<0.001$ |
| | RP | 16 | $r=0.9695$ | $P<0.001$ |
| All values | RE | 26 | $r=0.996$ | $P<0.001$ |
| | RP | 26 | $r=0.975$ | $P<0.001$ |

($\sim$10%) although there is a slight tendency for more marked dispersion for RP, in particular for low values of the non-specific binding.

### Discussion

The new method described here allows the simultaneous measurement of steroid receptors in a very small volume of cytosol. This has been made possible by combining the classical DCC technique with HPLC; the two additional steps are relatively rapid because only 3 hours are required for

**Table II** Intra-assay reproducibility of the micromethod

| | | *Results Five measurements of the same cytosol (cpm)* | | | *Means ± s.e.* | *Means of specific binding ± s.e.* |
|---|---|---|---|---|---|---|
| | Total binding | 172 | 185 | 171 | $\bar{m}=174\pm7.5$ | |
| | (T) | 168 | ND | | CV = 4.3% | $\bar{m}(T-NS)=146.8\pm7.7$ |
| RE | Non-specific binding | 27 | 28 | 28 | $\bar{m}=27.2\pm1.9$ | $=21.5\pm1.13$ fmol $mg^{-1}$ |
| | (NS) | 29 | 24 | | CV = 7% | CV = 5.2% |
| | Total binding | 574 | 619 | 565 | $\bar{m}=601.2\pm53.2$ | |
| | (T) | 687 | 561 | | CV = 8.8% | $\bar{m}(T-NS)=561.2\pm53.5$ |
| RP | Non-specific binding | 39 | 39 | 35 | $\bar{m}=40\pm4.8$ | $=82.1\pm7.8$ fmol $mg^{-1}$ |
| | (NS) | 48 | 39 | | CV = 11.9% | CV = 9.5% |

2 × 5 aliquots from one cytosol were used for the determination of the total and the non-specific binding as described in **Materials and methods**.
ND = not determined.
CV = coefficient of variation.

extraction of the steroids bound to the receptors, their separation by HPLC and their collection and quantification.

This work represents a novel application of HPLC which appears at present to be one of the most valuable techniques in clinical biochemistry (Elin, 1980). Connection of a continuous flux radioactivity detector at the outlet of the chromatograph may be used in order to allow automatic analysis. This study was centred on the two main receptors—RE and RP—since their evaluation has proved useful in the clinical management of breast cancer (Saez, 1981). However, the chromatographic conditions we have established also permit separation of dexamethasone from the two other hormones, thereby increasing the method's potential range of application. There is an inherent problem in the inclusion of androgen receptor measurements in this system because of the nearly identical affinity of metribolone (R1881) for androgen and progesterone receptors (Ojasoo & Raynaud, 1978).

The highly satisfactory correlation observed between the microassay and the DCC reference method implies that the thresholds of positivity established on the basis of the DCC method are not changed by the microassay, and thus should not alter the criteria used by clinicians in decision-making. Reproducibility tests revealed coefficients of variation close to 10%, indicating that the microassay is reliable, and obviating replicate measurements. Thus, with as little as 300 $\mu$l of cytosol, the protein concentration (50 $\mu$l) and the concentration of steroid receptors (2 × 100 $\mu$l) could be determined simultaneously. It is generally admitted that the lower limit for the protein concentration in cytosol is 1 mg $ml^{-1}$ if the DCC technique is to be successful (Poulsen *et al.*, 1979; Leclercq *et al.*, 1973). On this basis, samples of 20–30 mg can be analyzed by the microassay described here. This weight corresponds to the quantity of tissue obtained by a needle biopsy (Delarue *et al.*, 1981). This method is now used for the simultaneous measurement of RE and RP on biopsy specimens for *in situ* tumours in breast cancer patients (unpublished data).

The heterogeneity of steroid receptors within a mammary tumour is well established (Hawkins *et al.*, 1977; Poulsen, 1981). This variability can probably be explained by the histological disparity of mammary tumours (Gairard *et al.*, 1981), which means that it is advisable to couple each steroid receptor measurement on a biopsy specimen with microanatomical examination of a biopsy sample of a nearby site. For advanced breast cancer there are clinical indications preceding surgical intervention by combined radiotherapy and chemotherapy (Bruckmann *et al.*, 1979). Consequently, the biochemical characteristics of the cancer cells removed during surgery are theoretically no longer representative of the primary tumour prior to treatment. It is thus preferable to measure RE and RP on biopsy material prior to any treatment in order to evaluate the degree of hormone dependence. Certain studies suggest that RE status in a given patient changes as the tumour progresses (Osborne & McGuire, 1979) while other studies have indicated that this status is not modified when biopsy samples of the primary tumour or of subsequent metastases are examined (McGuire, 1980). To resolve this discrepancy, it would be interesting to conduct a sequential study based on measurements of RE and RP receptors on repeat biopsies of accessible lesions. The microassay presented here may constitute the analytical basis for such a programme.

### References

ALLEGRA, J.C., LIPPMAN, M.E., THOMSON, E.B. & 6 others. (1979). Distribution, frequency and quantitative analysis of Estrogen, Progesterone, Androgen and Glucocorticoid receptors in human breast cancer. *Cancer Res.*, **39**, 1447.

BRUCKMANN, J.E., HARRIS, J.R., LEVENE, M.D., CHAFFEY, J.T. & HELLMAN, S. (1979). Results of treating stage III carcinoma of the breast by primary irradiation therapy. *Cancer*, **43**, 985.

DELARUE, J.L., MOURIESSE, H., CONTESSO, G., MAY-LEVIN, F. & SANCHO-GARNIER, H. (1981). Microdosage des récepteurs hormonaux sur les forages biopsies dans les lésions et tumeurs mammaires humaines. *Biomedecine*, **34**, 153.

ELIN, R.J. (1980). Instrumentation in clinical chemistry. *Science*, **210**, 286.

GAIRARD, B., CALDEROLI, H., KEILING, R., RENAUD, R., BELLOCQ, J.P. & KOEHL, C. (1981). Cancer Cell counts and validity of steroid receptors determination in breast cancer. *Lancet*, **ii**, 1419.

HAWKINS, R.A., HILL, A., FREEDMAN, B., GORE, S.M., ROBERTS, M.M. & FORREST, A.P.M. (1977). Reproducibility of measurements of Oestrogen-receptor concentration in breast cancer. *Br. J. Cancer*, **36**, 355.

LECLERCQ, G., HEUSON, J., SCHOENFELD, R., MATTHEIM, W.H. & TAGNON, H.J. (1973). Estrogen receptors in human breast cancer. *Eur. J. Cancer*, **9**, 665.

MAGDALENAT, H. (1979). Simultaneous determination of Estrogen and Progestin receptors on small amounts of breast cancer (abstract). *Cancer Treat. Rep.*, **7**, 1147.

MCGUIRE, W.L. (1980). Steroid hormone receptors in breast cancer treatment strategy. *Rec. Prof. Horm. Res.*, **36**, 135.

MCGUIRE, W.L., DE LA GARZA, M. & CHAMNESS, G.C. (1977). Evaluation of estrogen receptor assays in human breast cancer tissue. *Cancer Res.*, **37**, 637.

OJASOO, T. & RAYNAUD, J.P. (1978). Unique steroid congeners for receptor studies. *Cancer Res.*, **38**, 4186.

OSBORNE, C.K. & MCGUIRE, W.L. (1979). The use of steroid hormone receptors in the treatment of human breast cancer: a review. *Bull. Cancer*, **66**, 203.

PICHON, M.F. & MILGROM, E. (1977). Characterization and assay of progesterone receptor in human mammary carcinoma. *Cancer Res.*, **37**, 464.

POULSEN, H.S. (1981). Estrogen receptor assay. Limitation of the method. *Eur. J. Cancer*, **17**, 494.

POULSEN, H.S., SCHULTZ, H. & BICHEL, P. (1979). Estrogen receptor determination on a fine needle aspiration from malignant tumor of the breast. *Eur. J. Cancer*, **15**, 1431.

SAEZ, S. (1981). Récepteurs des stéroides sexuels dans les tumeurs malignes. *Ann. Endocrinol.*, **42**, 306.

THIBODEAU, S.N., FREEMAN, L. & JIANG, N.S. (1981). Simultaneous measurement of estrogen and progesterone receptors in tumor cytosols with the use of $^{125}$I labelled Estradiol and of $^{3}$H-R5020. *Clin. Chem.*, **27**, 687.